# Chemical composition of processed bamboo for structural applications

**DOI:** 10.1007/s10570-018-1789-0

**Published:** 2018-04-23

**Authors:** Bhavna Sharma, Darshil U. Shah, Johnny Beaugrand, Emma-Rose Janeček, Oren A. Scherman, Michael H. Ramage

**Affiliations:** 1 Department of Architecture and Civil Engineering, University of Bath, Bath, UK; 2 Department of Architecture, Centre for Natural Material Innovation, University of Cambridge, Cambridge, UK; 3 FARE Laboratory, INRA, Université de Reims Champagne-Ardenne, 2 Esplanade Roland-Garros, 51100 Reims, France; 4 Biopolymères Interactions Assemblages (BIA), INRA, Rue de la Géraudière, 44316 Nantes, France; 5 Melville Laboratory for Polymer Synthesis, Department of Chemistry, University of Cambridge, Cambridge, UK

**Keywords:** Bleaching, Caramelisation, Chemical composition, Laminated bamboo, Treatment methods

## Abstract

Natural materials are a focus for development of low carbon products for a variety of applications. To utilise these materials, processing is required to meet acceptable industry standards. Laminated bamboo is a commercial product that is currently being explored for structural applications, however there is a gap in knowledge about the effects of commercial processing on the chemical composition. The present study utilised interdisciplinary methods of analysis to investigate the effects of processing on the composition of bamboo. Two common commercial processing methods were investigated: bleaching (chemical treatment) and caramelisation (hygrothermal treatment). The study indicated that the bleaching process results in a more pronounced degradation of the lignin in comparison to the caramelised bamboo. This augments previous research, which has shown that the processing method (strip size) and treatment may affect the mechanical properties of the material in the form of overall strength, failure modes and crack propagation. The study provides additional understanding of the effects of processing on the properties of bamboo.

## Introduction

Fibre reinforced composites are ubiquitous with uses in a variety of industries, including the automotive and infrastructure sectors. Synthetic fibres (such as glass, carbon, and aramid) embedded within a polymer matrix provide improved performance without increasing weight. While man-made composites dominate the market, there is growing interest in the use of renewable materials. Bamboo grows as a natural fibre composite with potential to be an alternative to conventional materials. The use of natural composites has multiple advantages, including the potential for being light-weight and offering environmentally-friendly routes to end-of-life disposal. For example, bio-based matrices are often degradable and are also used in energy recovery. Design and processing are key in the development of composites, whether synthetic or natural, to meet the requirements for performance.

**Fig. 1:**
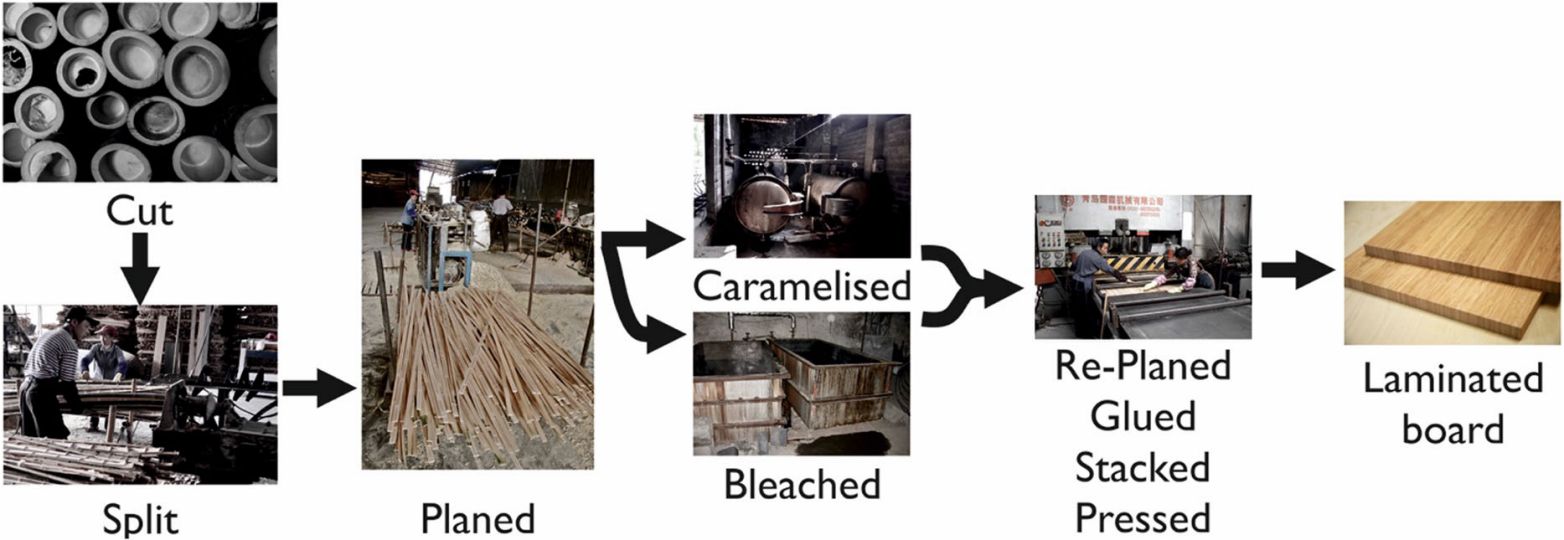
Commercial manufacturing methods for laminated bamboo (Reproduced with permission from Sharma et al. 2015)

Laminated bamboo is an engineered composite that is primarily used as a surface material (e.g. flooring, furniture, architectural detailing). The material is being increasingly explored for structural applications, however products are adopting existing manufacturing methods, as shown in Fig. 1. Although bamboo products are often compared to timber products, such as glue-laminated timber, the similarities are limited to them being plant-derived cellulosic materials. Reduced environmental impact is a major driver for use of laminated bamboo, with the cradle-to-gate production of a 19 mm single-ply laminated bamboo panel generating approximately 1.0 kg of carbon emissions per kg of product (kg CO_2_eq./kg), compared to 1.8 kg CO_2_eq./kg for steel and 11 kg CO_2_eq./kg for concrete (van der Lugt and Vogtländer 2015). For comparison, primary production of glue laminated timber generates approximately 0.8 kg CO_2_eq./kg (CES 2017). The additional processing required to manufacture the laminated bamboo panel increases the embodied carbon when compared to glue laminated timber.

A commercial board product, laminated bamboo sheets consist of raw bamboo material that undergoes one of two treatment processes: ‘bleaching’ or ‘caramelisation’ (Fig. 1). The choice of the process is dictated primarily by the colour preference for the surface material. In the bleaching process, the split and planed bamboo strips are bleached to a light yellow colour in a hydrogen peroxide bath at 70–80 °C (van der Lugt 2008). In contrast, the caramelisation process uses pressurised steam at approximately 120–130 °C (van der Lugt 2008), caramelising the sugars in the bamboo to obtain a deeper brown colour. The depth of colour differs based on the duration of treatment, which can vary from 4 to 8 h (van der Lugt 2008). Commercially, the caramelised colour is preferred for surface applications. However, the effects of processing on the mechanical properties, if any, are unknown (Sharma et al. 2015). These processing methods are also used as a preservation treatment, removing the sugars and starches (Liese 1980) in the culm to prevent biodegradation. The low microbial resistance of natural fibres leads to degradation issues before processing, which is avoided in laminated bamboo through processing of the material within 2–3 days of harvest.

Raw bamboo consists of cellulose, hemicellulose, lignin, ash and other extractives (Li 2004). The content varies between and within species and is dependent on the age of the culm, as well as the location along the height of the culm and within the culm wall (Li 2004). We note that the method of quantification itself may influence the amount of component quantified. For instance, with lignin Sluiter et al. (2010) track the evolution of the Klason protocol over the years and the difficulty to compare each other. Hatfield and Fukushima (2005) investigated the accuracy of lignin quantifications, by evaluating several methods. While we expect thermal treatment to alter the chemical composition of bamboo, the effects are yet to be characterised. Studies in existing literature primarily investigate compositional changes with thermal treatments in connection with the potential of bamboo for biomass, which are at temperatures substantially above those used in processing laminated bamboo, or when exploring non-structural applications of full-culm bamboo, such as for furniture. Bleaching with hydrogen peroxide is a process used in wood pulp and paper manufacturing, however the process has no precedent in manufacturing of structural bamboo materials.

Processing temperatures for plant-based materials are typically limited to 200 °C due to degradation of the fibres at higher temperatures (Jacob et al. 2005). In timber, thermal treatment is used to increase dimensional stability, reduce moisture content and improve durability. Thermal modification results in changes to the chemical structure and consequently mechanical properties (Tjeerdsma et al. 1998; Sundqvist et al. 2006; Poncsak et al. 2006; Kubojima et al. 2000; Hakkou et al. 2005; Campean et al. 2007; Bhuiyan et al. 2000). Some studies have reported that temperatures above 130 °C reduced the compressive strength of spruce, with the most significant degradation being the mass loss of hemicellulose, leading to a slight proportional increase in the relative ratio of cellulose and lignin at higher temperatures (Yildiz et al. 2006). The effect of thermal treatment on the chemical composition of wood also differs between species, with, for example, beech able to withstand temperatures up to 220 °C (Windeisen et al. 2007). Bamboos are wood-analogous materials in terms of their chemical make-up and similarly we envision the effects of preservation treatments to differ between species. The impact of steam treatment on the bending strength of hardwood species (such as black locust, oak, merbau, and sapupira) indicates that properties degrade above 100 °C (Varga and van der Zee 2008). To optimise the properties of the material, such as the dimensional stability and wettability, thermal treatment can be used, however the changes in chemical composition can affect the performance of the material in structural applications (Varga and van der Zee 2008).

Although biomass applications utilise temperatures above those used in laminated bamboo processing, studying the changes in composition occurring with thermal treatments is useful for understanding changes in physical properties of bamboo. Studies on bamboo charcoal have shown that the heat treatment of bamboo up to 200 °C degrades hemicellulose, and free water is generated due to chemical breakdown (Zuo et al. 2003). Duration of treatment also has an impact on the properties of bamboo with the strength and modulus of elasticity showing different tolerances to heat treatment, increasing up to 120 and 140 °C, before steadily decreasing (Zhang et al. 2013). The strength has also been shown to correlate with the mass loss due to thermal treatment arising from degradation of holocellulose and cellulose I (Zhang et al. 2013). Similar effects were noted in other species of bamboo with degradation occurring at increased temperatures and the effect of temperature was more significant than duration (Nguyen et al. 2012). The effect of thermal treatment increases dimensional stability, however excessive temperature and duration results in degradation at a micro-scale. The results suggest that heat treatment may be potentially tailored to alter the composition of the material for structural performance (Ramage et al. 2017). Our related work demonstrated significant differences in structural performance and failure modes (Sharma et al. 2015; Reynolds et al. 2016). The preservative treatments affected the load capacity and ductility before fracture in laminated bamboo dowel connections (Reynolds et al. 2016). The influence of the treatment methods on the microstructure of the material and crack propagation is not fully understood.

Further work assessed the influence of the strip size on the structural performance of the material (Penellum et al. 2018). Penellum et al. (2018) observed that the strip size, which results in an increase in the fibre volume fraction, correlates closely with the higher bending stiffness. The effect of the preservative treatment method on stiffness was unclear and more likely attributed to strip size (Penellum et al. 2018). The present study aims to clarify the effect of the changes in chemical composition of bamboo by two commercial processing methods (bleaching and caramelisation) on the resulting laminated bamboo products.

## Methodology

### Materials

The study compared the chemical composition of raw, bleached and caramelised Moso bamboo (*Phyllostachys pubescens*). The raw Moso bamboo is smoke treated but not additionally processed; samples were obtained from the full culm wall thickness, with the age of the culm unknown, however the material was sourced from the same supplier. The sample preparation removed the waxy exterior and pithy interior to obtain the culm material similar to that used in the processed product. The bleached and caramelised bamboo material were manufactured commercially (Supplier: Plyboo) and obtained from laminated bamboo boards, made from raw Moso bamboo between 3 and 5 years old at harvest (see Introduction for further details on processing). All three samples were prepared through grinding the material into a mix of sawdust and smaller particles. Each bamboo sample was analysed at a minimum in triplicate, and the mean values are given.

### Experimental methods

#### Biochemical composition

For the lignin and the carbohydrate content determination, samples were homogenised by grinding into a fine powder. The biomass composition process utilised 3 g of each sample type, which were milled in a centrifugal grinding mill (Retsch ZM100) equipped with a 0.2 mm sieve under liquid nitrogen environment. The carbohydrate identification and quantification were performed using HPAEC (ICS-5000^+^ DC, Dionex) following the procedure described elsewhere (Beaugrand et al. 2014). The neutral and acidic carbohydrates were determined from approximately 5 mg of powdered bamboo samples, which were subjected to hydrolysis in 12 M H_2_SO_4_ for 2 h at room temperature, and then diluted with distilled water to 1.5 M for 2 h at 100 °C. Samples are then filtered and injected into a CarboPac PA-1 anion exchange column (4 × 250 mm, Dionex). The monosaccharide composition was quantified using 2-deoxy-d-ribose as the internal standard, and using standard solutions of uronic acids (d(+)-galacturonic acid and d(+)-glucuronic acid) and neutral carbohydrates (l-arabinose, l-fucose, d-glucose, d-xylose, d-galactose, and d-mannose). Analyses were performed in three independent assays. The carbohydrate content, the sum of the monosaccharide amounts, is expressed as the percentage of the dry matter mass.

Prior to Klason lignin quantification, 2.5 g of each bamboo powder sample was subjected to a defatting step aimed at removing acid-insoluble extractives that may overestimate the Klason lignin amount. In the process, samples were subject to 6 h extraction by soxlhet (500 mL) with a laboratory grade (Sigma-Aldrich) ethanol-toluene (1/2 v/v) mixture in a chemical fume hood. The samples were then washed with ethanol (once) and then further with hot water (twice) and thereafter dried in an oven (Memmert, SingleDISPLAY 110) at 60 °C overnight. The lignin content was determined using the regular 72 wt% sulfuric acid Klason method, as described in the Technical Association of the Pulp and Paper Industry (TAPPI) Standard method T 222 om-02 (TAPPI 2002), where the acid-insoluble residue is the non-hydrolysable acid residue remaining after sulfuric acid hydrolysis. For each bamboo sample, the ground and defatted samples (3 replicates for each condition, precisely 0.5 g) were carefully immersed (to obtain good wettability of the samples) in 3.0 mL of 72% H_2_SO_4_ with a 20 mL graduated glass pipette, in a 20 mL beaker in fume hood and stirred every 15 min with a glass rod for a total of 2 h reaction at laboratory temperature (climatic lab 20 °C ± 2). The beakers were capped and then the contents were carefully transferred into a 500 mL Erlenmeyer flask and the beakers were meticulously rinsed with distilled water to avoid loss of particles, to a final volume of 115 mL in the flask (approx. 3% H_2_SO_4_).

The flask was then equipped with a reflux condenser (16 °C target temperature) and the suspensions boiled for 3 h with a hot plate (target temperature of 120 °C). The heating was turned off and the flasks were then let to cool down for 1 h over the heating plate. Next, the acid insoluble lignin were collected in a pre-weighed (after drying at 105 °C overnight) filtering Gooch glass crucible where an extra filter were deposit (Whatman^®^ qualitative filter, mesh of 11 µm). The transferred acid-insoluble lignin was then rinsed in the crucible with warm distilled water (50 °C, filled in a 100 mL measuring cylinder). The pH of the filtrate water was checked with a pH strip and was between 6 and 7 at the end of the rinsing; if it was acidic, more rinsing water was used. The remaining residues were dried at 105 °C for 20 h, and thereafter cooled in a desiccator prior to weighing. The ash measurements were performed after an additional 4 h at 500 °C. The Klason lignin percentages were calculated using the differences in the mass of the samples before and after ash measurements.

#### Chemical structure

Attenuated total reflectance Fourier transform infrared spectroscopy (ATR-FTIR) spectra were acquired (Perkin-Elmer, USA) with 16 scans taken for each sample with a resolution of 2 cm^−1^. A fine sawdust was scooped and packed onto the spectrometer. The spectral range was from 700 to 4000 cm^−1^. X-ray diffraction (XRD) analysis was carried out (Empyrean, PANalytical BV, Netherlands) at ambient temperature. A continuous scan was carried out for the angle range of 2θ = 5°–80° with data recorded every 0.2°. Crystallinity (C) was calculated using Eq. 1 (Park et al. 2010), where I_tot_ is the intensity at the primary (002) peak for cellulose I (at 2θ = 22°) and I_am_ is the intensity from the amorphous portion evaluated as the minimum intensity (at 2θ = 18.5°) between the primary (002) peak and the secondary (101) peak (at 2θ = 16°).

1 $$C = \frac{{I_{tot} - I_{am} }}{{I_{tot} }} \times 100$$

High resolution ^13^C cross-polarisation (CP) magic angle spinning (MAS) solid- state NMR (^13^C CPMAS NMR) analysis was performed on a 400 MHz spectrometer (Bruker Avance, USA) operating at 100.6 MHz using a 4 mm double resonance MAS probe spinning at 12.5 kHz. The chemical shifts were measured relative to tetramethylsilane via glycine as an external secondary reference with the Cα set to 43.1 ppm. Samples were packed in zirconia rotors, 4 mm in diameter, with Kel-F cap. The experiments were carried out using a 50–100% ^1^H ramped contact pulse with a contact time of 5000 µs and a proton power during contact of 3.5 dB. The ^13^C power level during contact was 8.7 dB. Spectra were collected for a total of 2048 scans. The recycle delay was 4 s and experiments were carried out at ambient temperature.

#### Hygrothermal properties

Differential Scanning Calorimetry (DSC; TA Instrument, USA) was performed on as-produced bamboo powders (i.e. no prior drying) to examine the influence of treatment on degradation and glass transition behaviour. The measurements were made under nitrogen flow (100 mL/min) from ambient to 600 °C with a constant heating rate of 10 °C per minute using an alumina crucible with a pinhole.

Dynamic Vapour Sorption (DVS) was used to obtain water adsorption isotherms (Hiden Isochema Ltd., UK). Water uptake was determined on small sticks of bamboo samples. The method used is described elsewhere (Placet et al. 2017) and was slightly modified as follows: the samples were first equilibrated at 95% relative humidity (RH), then a stepwise desorption setup was applied by decreasing the RH down to a dried state (0% RH), after which an adsorption cycle was performed. The cycle used approximately 5 mg of bamboo samples placed into the stainless steel nacelle of the microbalance, which was then placed hermetically in the double jacket reactor that was connected to a thermostated water bath. The temperature was set to 20 °C. The water uptake of the bamboo fibre was recorded at each equilibrium of the chosen RH values, and calculated as the water uptake at each equilibrium moisture content expressed as the dry matter of the sample (at 0% RH).

## Results and discussion

### Biochemical composition

The composition of the three samples is presented in Table 1. It is evident that processing affects the overall composition of the bleached and caramelised bamboo.

**Table 1:** Biomass composition of raw, bleached, and caramelised Moso bamboo

Monosaccharides % dry matter mean (SD)	Raw	Bleached	Caramelised
Fuc	0.02 (0.0)	0.02 (0.0)	0.01 (0.0)
Ara	0.9 (0.0)	1.0 (0.0)	0.7 (0.0)
Rha	0.1 (0.0)	0.0 (0.0)	0.0 (0.0)
Gal	0.4 (0.1)	0.3 (0.0)	0.2 (0.0)
Glu	42.2 (0.5)	36.4 (1.4)	34.8 (0.3)
Xyl	16.8 (0.4)	21.0 (0.5)	20.4 (0.1)
Man	0.3 (0.0)	0.2 (0.0)	0.3 (0.0)
GalA	0.5 (0.0)	0.5 (0.0)	0.4 (0.0)
GluA	0.1 (0.0)	0.1 (0.0)	0.1 (0.0)
Total	61.2 (0.8)	59.6 (2.0)	57.0 (0.5)
Xyl:Ara	18.7	21.0	29.1
Xyl:Glu	0.40	0.58	0.59
Klason Lignin	22.7 (0.14)	25.3 (2.43)	27.0 (0.12)

The principal hemicellulose present in bamboo is 4-*O*-methyl-*D*-glucurono-arabino-xylan (Maekawa 1976; Vena et al. 2010). The xylan contains the residues d-xylose (Xyl) and l-arabinose (Ara), as well as small fractions of l-galactose (Gal) and Glucuronic acid (GalA) (Maekawa 1976). We observed that Xyl, which is usually more sensitive to chemical degradation than d-glucose (Glu), does not decrease with processing, but rather increases in proportion. This is the case for both the bleaching and caramelisation processes. The relative loss of Glu may reflect loss of betaglucan (mixed linkage glucan), because it is less likely that the crystalline cellulose is destroyed by the procedures. The relative resistance to degradation of bamboo xylan may be related to its ability, like that of various xylans from other Poaceae (Beaugrand 2004; Beaugrand et al. 2004), to covalently bond with other xylan or lignins via hydroxycinnamic moieties (such as ferulic acids) to form a cross-linked network which limits chemical access. This behaviour would be similar to that of various xylans from the grass family (Beaugrand 2004; Beaugrand et al. 2004).

The degree of arabinose substitution (Xyl:Ara ratio) in bamboo differs from that observed in hardwoods and softwoods (Table 2). Xyl:Ara ratios of 15–20 have been previously reported for raw bamboo (Maekawa 1976; Shao et al. 2008). In comparison to bamboo, softwoods have relatively higher amounts of Ara (lower Xyl:Ara ratio), and hardwoods have fewer Ara residues (higher Xyl:Ara ratio) (Maekawa 1976; Timell 1973; Willför et al. 2005). As shown in Table 1, the processed materials indicate an increase in the Xyl:Ara ratio in comparison to the raw bamboo (18.7), with a slight increase in the bleached samples (21.0) and a marked increase in the caramelised material (29.1).

**Table 2:** Representative Xyl:Ara ratios in processed bamboo, Moso bamboo, and selected grasses, softwoods and hardwoods

Plant material	Xyl:Ara
Raw and processed bamboo (present study)	
Raw Moso bamboo	18.7
Bleached bamboo	21.0
Caramelised bamboo	29.1
Moso bamboo (Shao et al. 2008)	
*Phyllostachys pubescens* (Moso)	20.3
Grasses (Ford and Elliott 1987)	
*Digitaria eriantha* (Pangola)	6.6
*Setaria sp.*	7.7
*Hordeum vulgare* L. (Barley straw)	8.4
*Saccharum sp.* (Sugar cane)	10.3
Compression wood in softwoods (Timell 1973)	
*Abies balsamea* (North American Fir)	7.6
*Larix laricina* (American Larch)	4.6
*Piccea mariana* (Black Spruce)	7.3
*Pinus resinosa* (Norway Pine)	5.4
*Tsuga canadensis* (Eastern or Canadian Hemlock)	6.9
Selected heartwood in hardwoods (Willför et al. 2005)	
*Acacia mangium*	77.8
*Betula pendula* (Silver Birch)	44.3
*Eucalyptus globulus* (Tasmanian Bluegum)	24.8
*Fagus sylvatica* (European or Common Beech)	35.8
*Populus grandidentata* (American Aspen or White Poplar)	41.0
*Quercus robur* (European or English Oak)	28.0

In comparison to wood materials, the increase in the Xyl:Glu ratio with processing is attributed to the different structure of bamboo. The degradation and solubilisation of d-glucose, likely belonging to mixed linked betaglucans or amorphous cellulose, and the resistance of bamboo xylan to degradation (Beaugrand 2004; Beaugrand et al. 2004), may explain the observation. Xyl:Glu ratios for hardwoods have been shown to vary from 0.13 to 0.55 (Huang et al. 2016). Bamboo xylan is also characterised by a rather high degree of acetylation. The acetyl group content represents 6–7% of total bamboo xylan, compared to the 8–17% acetyl group content in xylan of hardwoods and 4–9% in glucomannan of softwoods (Vena et al. 2010). An observed difference between the bleached and caramelised materials is the release of acetates during caramelisation, which is likely to be a result of degradation of the acetyl esters.

Bamboo lignin comprises *p*-coumaryl units with guaiacyl, syringyl and *p*-hydroxyphenyl moieties, similar to grasses, or Poaceae plant lignin (Banik et al. 2017). Although bamboo lignins are richer in phenolic hydroxyl groups than wood lignins, and therefore more reactive during pulping (Vena et al. 2010), no substantial change in Klason lignin content was observed upon bleaching the raw bamboo (Table 1). This is likely because bamboo lignin is more resistant to bleaching than wood, as the former exhibits a higher degree of condensation (Salmela et al. 2008). In addition, our bamboos have been processed through ‘lignin-preserving bleaching’ under much milder conditions than ‘lignin-degrading bleaching’ (Ek et al. 2009). Consequently, while the bamboo bleaching process may lead to cell wall disruption and partial lignin oxidation (of chromophoric groups evidenced by the visual colour change), the lignin polymers are not solubilised or removed, but have been condensed and are still detectable.

#### Chemical structure

**Fig. 2:**
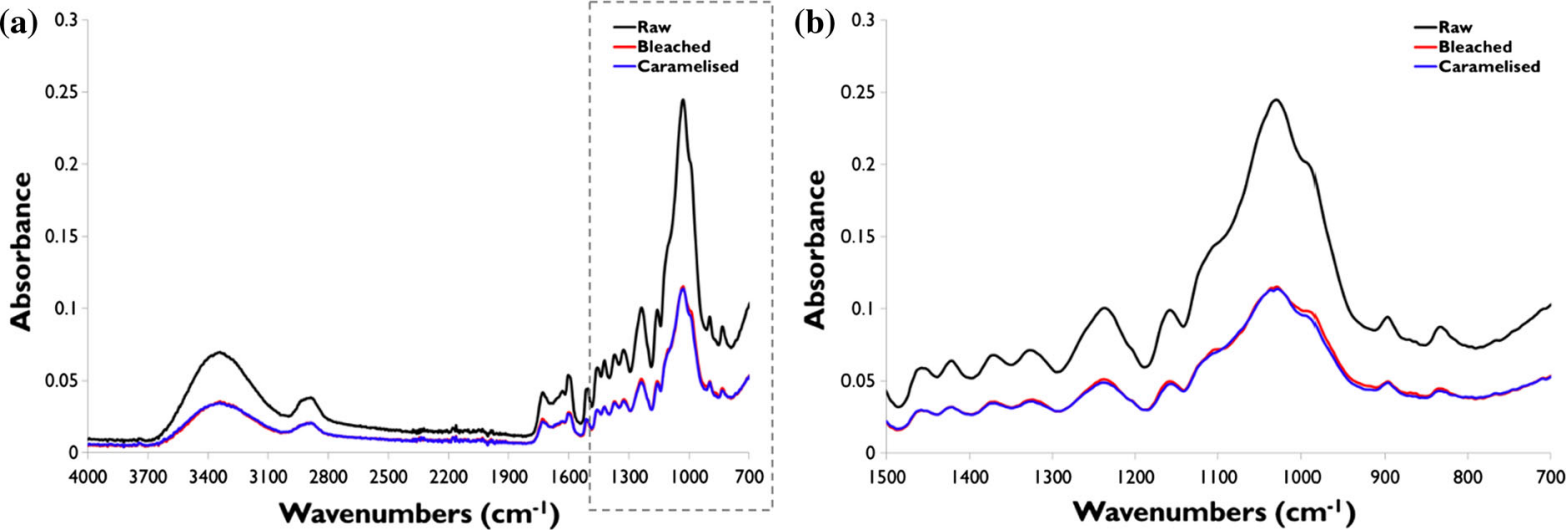
FTIR absolute **a** average absorbance spectra of raw Moso, bleached and caramelised bamboo with 700–1500 cm^−1^ region highlighted in **b**

The FTIR results are presented in Fig. 2. The absolute averaged spectra are plotted for each material. The results indicate that there are only subtle differences between the two treated samples. The observed shifts in the bleached material compared to the raw Moso bamboo are attributed to the bleaching process oxidising the aromatic rings of the phenolic groups in the lignin (1230 cm^−1^), as well as the hydroxyl groups in the polysaccharides (1047 cm^−1^). The latter would result in shorter cellulose chains and reduced crystallinity (Ramos et al. 2008; Douek and Goring 1976; Kishimoto et al. 2003). The C–O stretch at 1110 cm^−1^ has been attributed to loss of hydroxyl bonds and decomposition of hemicellulose (Zuo et al. 2003).

**Fig. 3:**
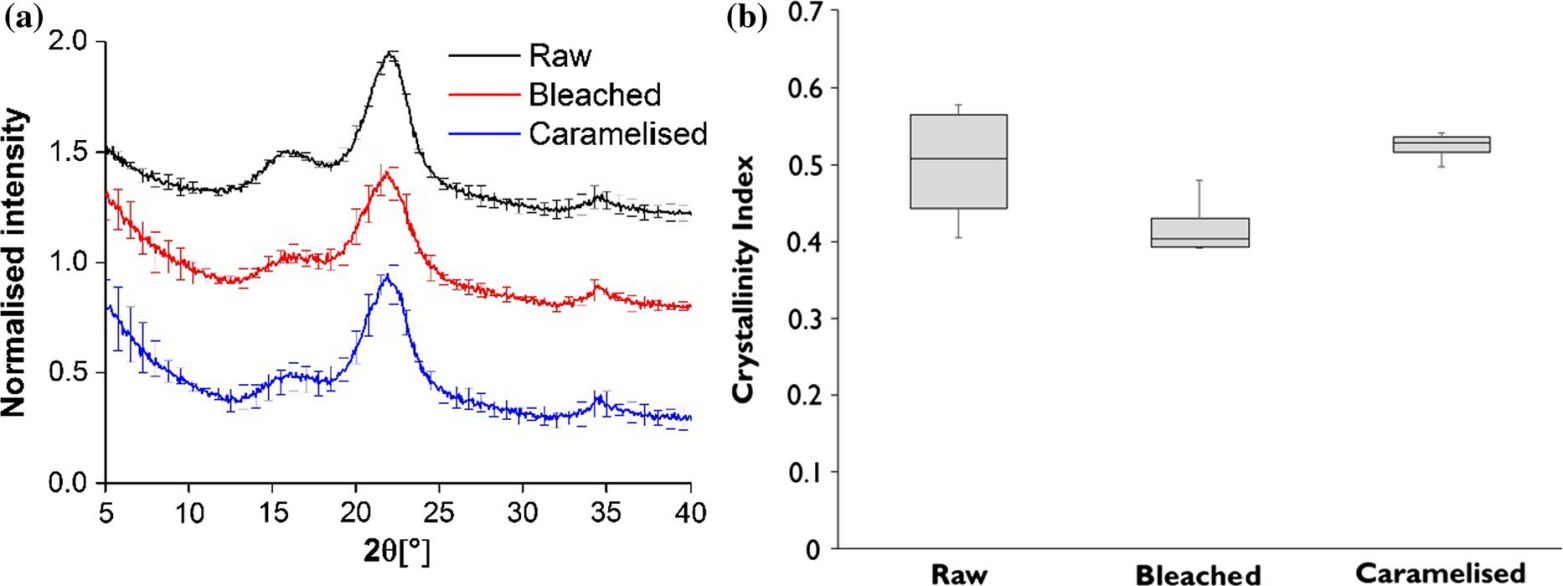
XRD was used to determine the crystallinity of raw Moso, bleached and caramelised bamboos. **a** XRD diffractograms of the three materials. **b** Calculated crystallinity index presented in box plots, which indicate the mean, 1st and 3rd quartiles, with the range shown in the whiskers

In comparison, the XRD spectra (Fig. 3a) revealed no noticeable shifts in any of the cellulose peaks nor any changes in peak shapes; all spectra reveal a crystalline (101) peak at 2θ = 16°, a (002) peak at 12.5°, and a (040) peak at 34.5°. However, peak intensities differed in the treated materials. Analysis of crystallinity index (Fig. 3b) reveals that while there is no statistically significant difference in the cellulose crystallinity of the raw and caramelised bamboos (*p* = 0.8, 2-tailed *t* test), the bleached bamboo has a substantially reduced crystallinity in comparison to both the raw (*p* = 0.10) and caramelised (*p* = 0.0004) materials. The reduced crystallinity of bleached bamboo is supported by the ATR-FTIR analysis. Analysis of the diffractograms and comparison with reference spectra of Cellulose I and II from (Takahashi and Takenaka 1987), it is evident that a transition from cellulose I to cellulose II is not observed, although this may be expected in a typical bleaching or alkali-treatment process of cellulose (Liu et al. 2012). This supports the idea that a ‘preserving bleaching’ process is used which eliminates the chromophoric groups in lignin, and while may lead to some depolymerisation, chain scission, and loss in crystallinity, is not harsh enough to substantially change the structure of cellulose from I to II.

Both bleached and caramelised bamboos undergo a steam-assisted drying step at 50–60 °C at the end of their treatment; the bleached bamboo is dried for 72 h, and caramelised bamboo for 240 h. The duration of steam-assisted drying processes is known to influence crystallinity of cellulosic materials, such as Eucalyptus and Spruce woods (Kong et al. 2017; Bhuiyan et al. 2000), and presumably also bamboo. Kong et al. observe that saturated steam at 100 °C initially (up to 4 h drying time) increases crystallinity of woods, due to moisture enabling higher mobility, reorientation and crystallisation of quasi-crystallised and amorphous regions. At higher drying times, increased chain scission reactions are proposed to increase amorphous fraction, and therefore reduce crystallinity. In our case, perhaps the lower drying temperatures (50–60 °C, rather than 100 °C) in saturated steam, extend these effects over longer durations.

**Fig. 4:**
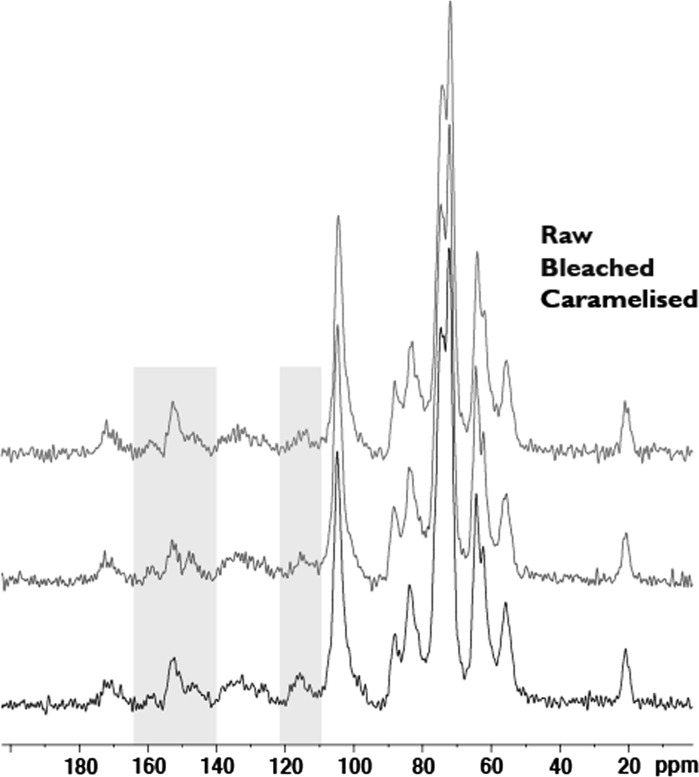
^13^C CPMAS NMR results for raw, bleached and caramelised bamboo

^13^C CPMAS NMR results indicate that for the bleached material signals in the 50–110 ppm region slightly decreased in intensity relative to the aromatics in the same sample when compared to the raw (Fig. 4). Comparing the raw and caramelised bamboo indicates that the processing results in changes in the 140–160 and 110–120 ppm regions of the spectra (highlighted in green). Peaks in this region are characteristic of aromatic or olefinic carbons, further analysis is required for full assignment.

### Hygrothermal properties

**Fig. 5:**
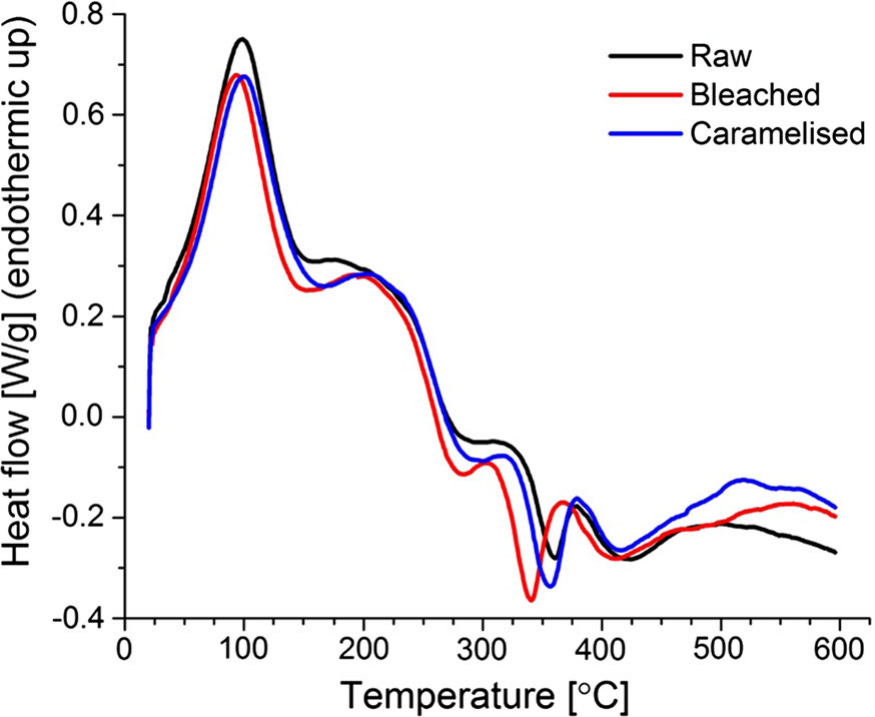
DSC results for raw, bleached and caramelised bamboo

In the DSC measurements (Fig. 5), the large endothermic peak at 100 °C for all materials is attributed to the removal of water. All endothermic and exothermic peaks at temperatures above 150 °C for bleached bamboo occur at 10–25 °C lower temperatures than that for raw and caramelised bamboo. The glass transition for the amorphous components is identified to be around 150–175 °C based on the inflection point. During pyrolysis, hemicelluloses and lignins volatilise (which is endothermic) and cellulose chars and leaves substantial residues (which is exothermic) (Yang et al. 2007). The exothermic peaks between 325 and 375 °C are associated with hemicellulose and lignin pyrolysis, while the endothermic peak between 350 and 400 °C is attributed to cellulose pyrolysis (Yang et al. 2007). The shifts in peaks suggests changes in hemicellulose and cellulose during the chemical bleaching process, making them more prone to pyrolysis, including shortening of chains and reduction in crystallinity confirmed through XRD measurements. This is also consistent with the reduction in signal intensity in the 50–110 ppm region observed by NMR. The hygrothermally treated caramelised bamboo shows a fairly similar profile to raw bamboos, with very minor shifts.

**Fig. 6:**
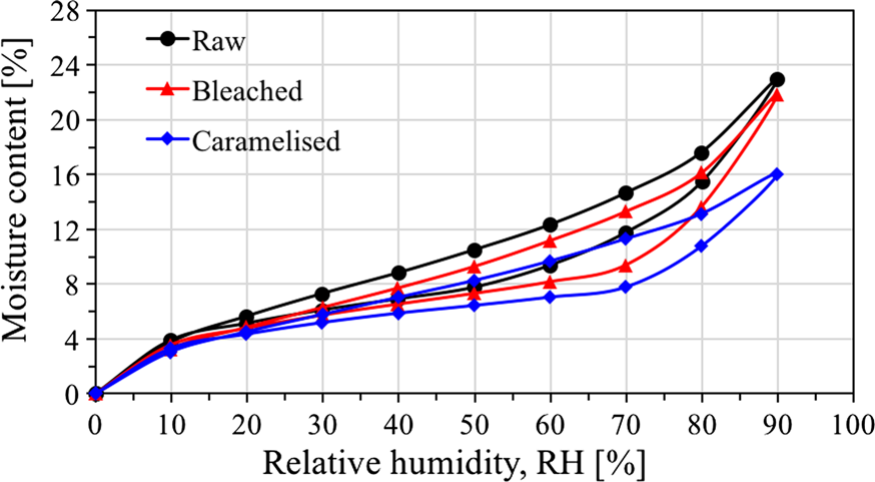
Moisture adsorption behaviour of the different bamboo materials

Dynamic vapour adsorption plots illustrating moisture absorption and desorption isotherms for the three bamboo materials are presented in Fig. 6. Typical type II sigmoidal profiles were observed, similar to that of plant fibres, depicting three distinct regions (Hill et al. 2009):

(i) Region A, up to ca 10% RH, where water is principally adsorbed through hydrogen bonding by amorphous cellulose and hemicellulose until saturation,
(ii) Region B, between 10 and 60% RH, where the porous cellular structure of bamboo enables adsorption of water into pores and micro-capillaries,
(iii) Region C, above 60% RH, where capillary condensation dominates and water molecules aggregate to form a fluidic, bulk phase.

The hysteresis response can be explained by the differing conditions in which adsorption and desorption take place (Hill et al. 2009). During adsorption, the bamboo material deforms by swelling and the micro-capillaries expand. However, during desorption, relaxation to the previous state is kinetically hindered due to changes in internal free volume, pore space (or micro-capillary space), surface area and structure.

The dynamic vapour adsorption results suggest that at any given relative humidity, the moisture content is higher in the raw material in comparison to the bleached and particularly the caramelised materials. This indicates that processing reduces adsorption capacity, which is further observed in the final products with the raw, bleached and caramelised materials having an average equilibrium moisture content of 10, 8 and 6%, respectively. In addition, the processed materials also show a pronounced hysteretic response. For example, hysteresis for the raw bamboo peaks to 3.0 at 60% RH, but is as high as 3.9 and 3.5 at 70% RH for the caramelised and bleached materials, respectively. This is substantially higher than the 1.5–3% peak hysteresis typically recorded for plant fibres (Hill et al. 2009). In general, high amorphous polysaccharide content, particularly amorphous cellulose and hemicellulose, may be responsible for higher water accessibility and moisture uptake. Both processed materials have an insubstantially reduced glucose content (Table 1), arguably belonging to cellulose (Maekawa 1976). Comparing the bleached and caramelised bamboo, we see greater effects on the caramelised products that are attributed to the removal of the hydroxyl groups, rather than the aromatics and phenolic groups in the bleached material.

In addition, differences in microstructure and micro-scale porosity also influence moisture capacity. The density of raw bamboo is lowest (at 562 kg/m^3^), followed by bleached bamboo (644 kg/m^3^) and caramelised bamboo (686 kg/m^3^). This suggests that untreated bamboo would have the highest porosity content (Eq. 2) at 62.5%, with the porosity content of bleached bamboo at 57.1% and of caramelised bamboo at 54.3%

2 $${\text{Porosity content}} = \left( {1 - {\text{raw material density}}/{\text{cell wall density}}} \right) \times 100$$

where cell wall density is assumed to be 1500 kg/m^3^ (Shah et al. 2016).

## Conclusions

The presented study investigated the effects of processing on the biochemical composition and chemical structure of raw Moso bamboo. The results indicate that the bleaching process results in a more pronounced degradation of the lignin in comparison to the caramelised bamboo. Subtle changes to the lignin composition and structure may have larger global effects in the form of fracture and crack propagation, however this is yet to be fully characterised. In general, caramelisation may have positive benefits for global material properties and durability of bamboo. This is further supported by previous experimental studies in which the bleached material often had a lower strength in comparison to the caramelised material. A better understanding of the effects of processing would help to elucidate the properties of bamboo and various derivative materials in structural applications.
